# Protocol for a systematic review of reductions in therapy for children with low-risk febrile neutropenia

**DOI:** 10.1186/2046-4053-3-119

**Published:** 2014-10-21

**Authors:** Jessica E Morgan, Lesley Stewart, Robert S Phillips

**Affiliations:** 1 Centre for Reviews and Dissemination, University of York, York, UK

**Keywords:** Fever, Neutropenia, Cancer, Children, Outpatient

## Abstract

**Background:**

Febrile neutropenia is a common complication of therapy in children with cancer. Some patients are at low risk of complications, and research has considered reduction in therapy for these patients. A previous systematic review broadly considered whether outpatient treatment and oral antibiotics were safe in this context and concluded that this was likely to be the case. Since that review, there has been further research in this area. Therefore, we aim to provide a more robust answer to these questions and to additionally explore whether the exact timing of discharge, including entirely outpatient treatment, has an impact on outcomes.

**Methods/design:**

The search will cover MEDLINE, MEDLINE In-Process & Other Non-Indexed Citations, EMBASE, CDSR, CENTRAL, LILACS, HTA and DARE. A full search strategy is provided. Key conference proceedings and reference lists of included papers will be hand searched. Prominent authors/clinicians in the field will be contacted. We will include randomised and quasi-randomised controlled trials along with prospective single-arm studies that examine the location of therapy and/or the route of administration of antibiotics in children or young adults (aged less than 18 years) who attend paediatric services with fever and neutropenia due to treatment for cancer and are assessed to be at low risk of medical complications. Studies will be screened and data extracted by one researcher and independently checked by a second. All studies will be critically appraised using tools appropriate to the study design. Data from randomised controlled trials (RCTs) will be combined to provide comparative estimates of treatment failure, safety and adequacy. Information from quasi-randomised trials and single-arm studies will provide further data on the safety and adequacy of regimes. Random effects meta-analysis will be used to combine studies. A detailed analysis plan, including assessment of heterogeneity and publication bias, is provided.

**Discussion:**

This study will aim to specifically define the features of a low-risk strategy that will maintain levels of safety and adequacy equivalent to those of traditional treatments. This will both inform the development of services and provide patients and families with data to help them make an informed decision about care.

**Systematic review registration:**

PROSPERO CRD42014005817

## Background

Febrile neutropenia describes the presence of fever, representing infection, in a patient who has poor immunity due to low neutrophils. It is the commonest life-threatening complication of treatment of children with cancer [1]. It occurs in around a third of episodes of neutropenia, at a rate of 0.75 episodes per 30 days at risk [2]. A large number of patients with febrile neutropenia have no significant sequelae of the condition, whilst a smaller number are at high risk of medical complications including organ failure and death.

Standard care in the UK usually involves intravenous (IV) antibiotics and at least 48 h as an inpatient [3]. However, research has begun to focus on whether treatment can be reduced safely for those patients who are considered to be at low risk of complications of febrile neutropenia [1]. This may provide benefits of improved quality of life, reduction in hospital-acquired infection, cost savings and reduced pressures on the health service [4-7]. However, any reduction in therapy must be both safe and effective to justify a change from current practice.

A previous systematic review compared two aspects of the reduction of therapy [8]. It compared outpatient treatment with inpatient treatment, and oral antibiotics with IV therapy. It included only low-risk patients as defined by individual study protocols, recognising that this is a heterogeneous group.

The review found that treatment failure was as likely to occur in inpatients compared with outpatients and that inpatients were more likely to have alterations made to their antibiotic regimes. Within the studies included, two low-risk patients died—they had both been treated as inpatients. They found no increase in medical complications with oral therapy. In particular, there were no deaths on oral therapy, and no increase in readmission to hospital or in treatment failure, including the need to modify the antibiotic regime.

Although this provided essential information about the broad concepts of outpatient and oral therapy for febrile neutropenia, it involved combining data from very different groups and thus lost some of the nuanced information from original trials. This is particularly important when considering timing of discharge as further information is required to know precisely when discharge should be advised. Furthermore, the review was restrictive in its search strategy, excluding non-English language papers, using few databases and having no grey literature searching. In addition to these limitations, important further work has since emerged in this area. In particular, one trial looked specifically at early step-down from intravenous antibiotics given as an inpatient to oral outpatient treatment within 22 h of presentation with febrile neutropenia [9].

Meanwhile, a recent Cochrane review looking at both adult and paediatric patients with febrile neutropenia found that oral therapy was an acceptable alternative to intravenous [10]. A subgroup analysis of paediatric studies found no difference in treatment failure rates within this population, although this included exclusively randomised controlled trials, of which there were only eight identified in children. Furthermore, it did not examine specifically the issue of location of treatment. Our systematic review, which will also include studies using observational methods, aims to provide further depth and clarity to these findings.

Given all of these issues and the high likelihood of other new and relevant studies, we decided that a new systematic review should be performed. Our review will have more focused aims, particularly in defining the most appropriate time of discharge in these patients, and will have a new and more thorough search strategy.

### Aims, objectives and overview of approach

This systematic review aims to provide an up-to-date and robust assessment of the role of the route of antibiotic administration and the location of treatment in the management of low-risk febrile neutropenia in children being treated for cancer. We will aim to define the overall success (or failure) of each treatment regime in resolving an episode of febrile neutropenia without complications. Furthermore, we intend to explore the main two components of this overall impression—that is safety and efficacy—for each potential regime. In particular, we will explore the timing of discharge (before 24 h, 24–48 h or after 48 h), including the role of entirely outpatient treatment. This will provide more detailed information about how low-risk services might be designed.

Finally, we understand that there may be concern regarding reduction of therapy from patients, their parents and the healthcare professionals caring for them. This systematic review will therefore collect data on rates of declined consent, where this has been reported, as a way of gaining insight into the potential acceptability of these approaches.

## Methods/design

This systematic review will be undertaken following guidelines from Cochrane [11] and CRD [12].

### Search and retrieval strategy

The search strategy focuses on febrile neutropenia and the interventions of antibiotics and early discharge, with a paediatric filter (see Additional file 1: Appendix 1). The antibiotic search terms were the same as those used in the development of the NICE clinical guideline for neutropenic sepsis and reflect the antibiotics that have been commonly used to treat febrile neutropenia, either previously or currently, within the UK [13].

The following electronic sources will be searched: MEDLINE, MEDLINE In-Process & Other Non-Indexed Citations, EMBASE, CDSR, CENTRAL (via the Cochrane Library), LILACS, HTA and DARE. The full MEDLINE search strategies are included in Additional file 1: Appendix 1.

Conference proceedings of the RCPCH (Royal College of Paediatrics and Child Health), SIOP (International Society of Paediatric Oncology), ASPHO (American Society of Pediatric Hematology/Oncology), ASCO (American Society of Clinical Oncology) and ICAAC (Interscience Conference on Antimicrobial Agents and Chemotherapy) meetings will be hand searched for relevant abstracts. Reference lists of relevant systematic reviews and included articles will also be reviewed.

Authors of relevant studies and prominent clinicians within the field will be contacted as time allows seeking further studies, as this is likely to be a poorly indexed area of biomedical research (see Additional file 1: Appendix 2).

Published and unpublished studies will be sought and no language restrictions applied. The latter is important because we suspect that there may be a number of studies that have been performed in Spain, Portugal and South America as these areas have active research in paediatric oncology. Non-English language studies will be translated if this is possible within 3 months of running the searches. This time limit will ensure that the results of this review are available to inform further aspects of an overarching multi-methods PhD project.

### Screening for eligibility

One reviewer will screen the title and abstract of all studies for inclusion. A second reviewer will independently screen a sample of 1,000 papers or 10% of those identified through the search, whichever is greater. The kappa statistic for agreement will be calculated, and if this shows significant disagreement (*κ* <0.4), all other titles and abstracts will be screened by a second reviewer. Where it is not possible to identify whether a study should be included from the title and abstract, then the full text of the paper will be sought and then assessed using the study eligibility form (see Additional file 1: Appendix 3). All full-text assessments will be performed by two reviewers. Disagreements regarding which studies to include will be resolved by consensus or, if this proves impossible, by recourse to an independent adjudicator.

### Inclusion and exclusion criteria

Studies will be included in the review if they meet the following criteria:

#### Study design

Outcomes of interest will be explored using both comparative and non-comparative data. Randomised controlled trials (RCTs) will provide the most reliable evidence on effectiveness and will also aim to give clarity on the role of timing of discharge in safety and adequacy. However, we anticipate that the number of RCTs in this area will be small.

Furthermore, there is benefit in knowing the absolute numbers of patients experiencing failures in safety and adequacy when treated with oral antibiotics on an outpatient basis. This can be obtained using non-comparative data from both single-arm studies and the separate arms of RCTs.

We will include RCTs and prospective single-arm studies. Quasi-randomised trials will be eligible for inclusion provided the methods of allocation to treatment groups are clearly described. Randomised controlled trials will be used for comparative analyses. Quasi-randomised trials will be analysed separately, but where they are assessed to be similar to the RCTs then the two groups will be combined. Where this combination is performed, this will be clearly stated in the report. Individual arms of the trials and prospective single-arm studies will be used for the non-comparative analyses.

Retrospective studies will be excluded. Studies that enrolled participants ≥24 h after initial empiric treatment will be excluded.

#### Population

The population will include children or young adults (aged less than 18 years) who attend paediatric services with fever and neutropenia secondary to treatment for cancer and who are assessed to be at low risk of medical complications.

We will include studies that enrolled only children. Studies in which the majority (defined as >80%) of patients are less than 18 years old will be included, even if those patients are not reported separately. This reflects the fact that some paediatric studies may include a small number of young adults who have malignancies and physiology similar to the paediatric cohort being investigated and who are therefore treated within teenage and young adult services by paediatricians. Such studies designed for the paediatric group that have recruited small numbers of young adults do not generally recruit from the older adult population, in which the causes and outcomes of febrile neutropenia are likely to be different. Studies of adults which report data for patients less than 18 years old will be included, if outcome data for children are reported separately. Studies which include >20% of adult populations and those which include mixed age populations with no details on age distribution will be excluded.

To be included, details of the risk stratification rule used must be available, but there will be no eligibility restriction concerning the definitions of fever and neutropenia or the stratification rule used. These are likely to vary across studies reflecting variations in current clinical practice [6]. Studies of multiple risk groups will be included, if data for low-risk patients (as defined by study protocols) can be extracted separately.

#### Interventions and comparators

We are aware that studies have included a variety of treatment regimes, including inpatient IV therapy, outpatient IV therapy and oral outpatient therapy [8]. This creates a challenge as there are two components to the comparison—the route of administration of the antibiotics (IV or oral) and the location of the patient’s treatment (outpatient or inpatient)—which could be correlated in individual studies. In particular, oral antibiotics and outpatient treatment are likely to be used together and it may be difficult to establish whether any differences in outcome are related to the route, the location of treatment or both. The considerable intermeshing of these two issues within the literature justifies the use of a single review to attempt to address the various options in reduction of therapy to these patients.

Prospective single-arm studies must examine the features of route of administration and/or location of treatment as the primary aim of the study. We anticipate that these studies will therefore describe oral therapy and/or outpatient location. Studies examining different antibiotics given by the same route and in the same location will be excluded.

To be included, a study must have investigated for one or more of the following interventions:

(i) Location of treatment:

We will include studies that compare outpatient with inpatient (less than 8 h in hospital) care. The location of treatment may change at any point during the episode of febrile neutropenia, but the time of change must be clearly reported.

Justification for using an 8-h cut-off is a practical one. The time taken to review a patient, ensure they are eligible for outpatient treatment and then prescribe and obtain any medications to take home may be up to 8 h. Any period longer than this could reasonably be called admission to hospital.

(ii) Route of antibiotic administration:

We will include studies that compare oral with IV antibiotics. The route of administration may change at any point during the episode of febrile neutropenia but the time of change must be clearly reported.

Where patients receive a single dose of IV antibiotics followed by a course of oral antibiotics, this will be considered to be an entirely oral course. This is to allow for studies in which IV antibiotics are administered whilst awaiting the results of blood tests to confirm low risk status, but once the patient is identified as low risk, they are started on oral antibiotics.

The sheer variety in the antibiotics used for paediatric febrile neutropenia will add complexity. Both the previous review by Manji et al. and the recent NICE guideline found numerous different antibiotic regimes studied, in both adult and paediatric protocols [8,14]. It is worth noting however that although the specific antibiotics used are varied, the coverage of these antibiotics is certainly less so. Thus, differences between regimes are more likely to be related to the route of administration, including absorption and dosing, than the specific antibiotic used.

#### Outcomes

There will be three primary outcomes in this review: treatment failure, safety and adequacy. Treatment failure is a composite outcome for various reasons. Firstly, this is in line with multinational guidelines which have recommended that the primary outcome of studies into febrile neutropenia should be such a composite measure [15]. The rationale for this approach is that this is likely to be the information that patients and clinicians combine when making decisions about choice of care; thus, it is the most clinically relevant outcome for those involved in paediatric haematology and oncology services. In addition, from a practical point of view, the studies which are likely to be included have generally reported composite outcomes, rather than individual medical complications [16].

We will also address two important parts of this composite outcome separately. This is because each informs separate aspects of service transformation and because different sources of evidence will be considered for each (primarily because anticipated low numbers of adverse events mean that we will need to look beyond comparative studies). Exploration of safety will consider the frequency of medical complications, in particular admissions to critical care services or death. Adequacy will relate to the ability of a treatment protocol to result in resolution of the episode of febrile neutropenia, without change in antibiotic or location of the patient. Although these outcomes might be seen as different ways of measuring a similar problem, from a clinical point of view, they are important for different reasons. Knowledge about the safety of a strategy is essential to be able to consider its use at all, whilst adequacy allows services to plan appropriately for potential readmissions or changes in treatment associated with changing to a new low-risk strategy.

To be included, a study must have recorded and data be available for one or more of the following outcomes:

##### Primary outcomes

1. Treatment failure at 30 days. This will include persistence, worsening or recurrence of fever/infecting organisms, new infections, readmission, admission to critical care services or death during treatment. For the primary outcome, modification of antibiotics will also indicate treatment failure.

We acknowledge that each study is unlikely to select an outcome that completely fits the definition given above. Therefore, the composite outcome that each individual study selects will be recorded within the data collection stage of this systematic review. The selection of 30 days as a cut-off for this outcome is for both clinical and practical reasons. Clinically, most periods of neutropenia last for less than 30 days and, indeed, most chemotherapy regimes are based on cycles of around 3 weeks for this reason. Thus, individual episodes of febrile neutropenia are generally limited within this time frame. Related to this, and on a more practical note, studies in the area of febrile neutropenia usually report outcomes at 28 to 30 days.

2. Safety: number of medical complications, defined as admissions to critical care services or death.

3. Adequacy: ability of a treatment protocol to result in resolution of the episode of febrile neutropenia, without change in antibiotic or location of the patient.

##### Secondary outcomes

The secondary outcomes will include treatment failure with modification of antibiotics excluded, time spent in hospital, adverse events leading to antibiotic discontinuation, 30-day overall mortality and infection-related mortality and the individual components of the primary composite outcome.

### Data extraction and assessment of risk of bias

Data will be extracted by one researcher using a standardised data extraction form (see Additional file 1: Appendix 4) and independently checked by a second. If the data to be extracted is unclear, authors will be contacted for further information. If there is no response, a further attempt to make contact will be made a fortnight later. If there is no response after a further 4 weeks, the data will be presumed unavailable.

The risk of bias in each study will be assessed using an appropriate tool, dependent on the design of the original study (see Additional file 1: Appendix 5). Assessment of randomised controlled trials will be based on the Cochrane risk of bias [11]. Single-arm studies will be assessed using the tool written by Hayden and used by NICE for prognostic studies [17].

### Methods of analysis/synthesis

The study characteristics and quality assessments will be described narratively and represented in tabular form. A full list of anticipated statistical analyses can be found in Additional file 1: Appendix 6. Any *post hoc* analyses performed outwith those defined in this protocol will be clearly identified as such in the presentation of results.

(1) Comparative analysis

Randomised controlled trials and quasi-randomised trials will be analysed as separate groups. These will be combined if they include similar patients and methods, so as to give an estimate of effect in all studies.

Interventions will be analysed separately by location of treatment and by route of antibiotic administration.

Odds ratios will be calculated for treatment failure at 30 days, safety and adequacy for each individual study, assessing the route of antibiotic administration and location of treatment separately. These odds ratios will then be combined using a random effects model, given the anticipated clinical heterogeneity. Forest plots will be presented for each outcome.

Weighted mean differences will be calculated for rates of treatment failure, safety and adequacy of both oral antibiotics compared with IV, and outpatient compared with inpatient treatment.

Results will be expressed in both relative (odds ratios) and absolute terms (weighted mean difference).

(2) Wider exploration of safety and adequacy

Safety and adequacy outcomes will be explored using data derived from single-arm studies and from the individual arms of comparative studies.

Weighted averages for treatment failure, safety and adequacy of different routes of administration and locations of treatment will be reported separately.

A weighted average of the readmission rate in outpatient treatment arms will also be calculated.

(3) Exploration of consent to randomisation

We will use the data from all included studies (RCTS, prospective cohorts and prospective single-arm studies) combined using a random effects model to report a weighted average of declining to consent for reduction in therapy studies.

### Heterogeneity

Heterogeneity will be examined by visual inspection of forest plots and using the chi-squared test of heterogeneity (recognising that this test has low power in meta-analyses of multiple small studies which this systematic review is likely to include). The *I*^2^ test will be used to quantify the degree of heterogeneity that is not due to chance. The tau^2^ statistic will be calculated to assess the between-study variance of true effect.

### Subgroup analyses

#### Time of discharge

Studies will be grouped according to their definition of early discharge to explore whether the benefits and risks of early discharge are affected by different time periods

•before 24 h,

•24–48 h,

•after 48 h.

These time periods were selected as they represent the frequency of medical ward rounds, at which the patient is most likely to be reviewed and potentially discharged. Assessing these time points may provide particularly salient clinical information as to a specific point at which consideration of early discharge might be most appropriate. Where studies provide a range of times at which early discharge might be considered, for example 8 to 22 h as in the SPOG study, the earliest time at which discharge could be considered will be the time used to categorise the study [9].

#### Risk stratification tool

There are a large number of different risk stratification tools in current use (25 in the most up-to-date systematic review) which creates a potential source of clinical difference between studies [18]. For those rules that have been considered in more than one study, studies will be grouped by the specific risk stratification tool used and subgroup analysis performed.

#### Time of risk assessment

The time at which the risk assessment is made may affect which patients are eligible to be discharged and may affect results from the study. When risk assessment is performed later in the episode of febrile neutropenia, for example at 24 h rather than at admission, then more information is likely to be available for this assessment, such that the risk of misclassifying a patient as low risk when they are in fact high risk is reduced. Therefore, the outcomes for patients in whom the risk assessment is performed later may be better than those who are risk stratified at presentation. This hypothesis will also be assessed by subgroup analysis.

•Risk stratified within 8 h of presentation

•Risk stratified between 8 and 24 h

•Risk stratified after 24 h

### Sensitivity analyses

Potential areas of heterogeneity that will be explored using sensitivity analyses include the inclusion/exclusion of studies reported as conference abstracts, restricting comparative analyses to randomised controlled trials only, risk of bias assessed by components (particularly selection and attrition bias), the use of fixed effects rather than random effects meta-analysis, definitions of fever and neutropenia used by the study, the definition of treatment failure used by the study, the specific antibiotics used in each arm of the studies, and the location of the study, which provides information on both standards of care and the likely micro-organisms involved in febrile neutropenia.

### Publication bias

Publication bias within this area may be relatively high, given that studies in children and young people are often small and may be difficult to recruit into. These studies are more likely to produce non-significant results and are thus at risk of positive result and time lag biases [19]. However, these studies may be of greatest interest in this particular research question.

In recognition of this, along with thorough searching, the data analysis phase will explore the risk of publication bias. A contour-enhanced funnel plot will be created for each primary outcome, showing the value for each study (i.e. the rate of treatment failure, medical complications or efficacious treatment) plotted against sample size. Formal statistical testing for publication bias will be performed using Harbord and Peters tests, as the primary outcome is binary. Any potential bias will be investigated with a cumulative forest plot to identify the impact of smaller studies on the results.

### Methods of dissemination

The results of this review will be written for publication in a scholarly journal following the PRISMA reporting guidelines as closely as possible [20]. Areas of uncertainty and suggestions for further research will be outlined within the final report. Lay and professional summaries of the project will also be produced and be available on the CRD’s website. The lay summary will be offered for inclusion in the newsletter produced by Candlelighters, the children’s cancer charity that is supporting this work.

## Discussion

This study will aim to specifically define the features of a low-risk strategy for paediatric febrile neutropenia that will maintain levels of safety and adequacy equivalent to those of traditional treatments. This will both inform the development of services and provide patients and families with data to help them make an informed decision about care.

## Abbreviations

IV: intravenous; NICE: National Institute for Health and Care Excellence; PRISMA: Preferred Reporting Items for Systematic Reviews and Meta-Analyses; SPOG: Swiss Paediatric Oncology Group.

## Competing interests

The authors declare that they have no competing interests.

## Authors’ contributions

JM designed the study in collaboration with other authors and obtained funding for it as part of her PhD fellowship. She developed the search strategies in collaboration with an information specialist and registered and drafted the protocol. She will coordinate the study and act as the principal reviewer. RP conceived the study and helped to inform the study design and revise the protocol. LS helped to inform the study design and revise the manuscript. All authors read and approved the final protocol.

## Supplementary Material

Additional file 1 **Appendix 1: Search strategy. Appendix 2**: Email to elicit unpublished studies. **Appendix 3**: Study eligibility decision form. **Appendix 4**: Data extraction tool. **Appendix 5**: Assessment of risk of bias. **Appendix 6**: Anticipated statistical analyses. Click here for file

## References

[B1] Basu SK Fernandez ID Fisher SG Asselin BL Lyman GH Length of stay and mortality associated with febrile neutropenia among children with cancer J Clin Oncol 2005 23 7958 7966 10.1200/JCO.2005.01.6378 16258096

[B2] Castagnola E Fontana V Caviglia I Caruso S Faraci M Fioredda F Garrè ML Moroni C Conte M Losurdo G Scuderi F Bandettini R Tomà P Viscoli C Haupt R A prospective study on the epidemiology of febrile episodes during chemotherapy-induced neutropenia in children with cancer or after hemopoietic stem cell transplantation Clin Infect Dis 2007 45 1296 1304 10.1086/522533 17968824

[B3] Bate J Gibson F Selwood K Skinner R Phillips B Chisholm JC A reaudit of current febrile neutropenia practice in UK paediatric oncology centres prior to implementation of NICE guidance Arch Dis Child 2013 98 315 316 10.1136/archdischild-2013-303810 23467661

[B4] Teuffel O Cheng S Ethier MC Diorio C Martino J Mayo C Wing R Sung L Alibhai SMH Health-related quality of life anticipated with different management strategies for febrile neutropenia in adult cancer patients Support Care Cancer Off J Multinatl Assoc Support Care Cancer 2012 20 2755 2764 10.1007/s00520-012-1397-8 22350594

[B5] Sung L Aplenc R Alonzo TA Gerbing RB Lehrnbecher T Gamis AS Effectiveness of supportive care measures to reduce infections in pediatric AML: a report from the Children’s Oncology Group Blood 2013 121 3573 3577 10.1182/blood-2013-01-476614 23471307 PMC3643758

[B6] Mustafa MM Aquino VM Pappo A Tkaczewski I Buchanan GR A pilot study of outpatient management of febrile neutropenic children with cancer at low risk of bacteremia J Pediatr 1996 128 847 849 10.1016/S0022-3476(96)70339-6 8648546

[B7] Teuffel O Amir E Alibhai SMH Beyene J Sung L Cost-effectiveness of outpatient management for febrile neutropenia in children with cancer Pediatrics 2011 127 e279 286 10.1542/peds.2010-0734 21220399

[B8] Manji A Beyene J Dupuis LL Phillips R Lehrnbecher T Sung L Outpatient and oral antibiotic management of low-risk febrile neutropenia are effective in children–a systematic review of prospective trials Support Care Cancer Off J Multinatl Assoc Support Care Cancer 2012 20 1135 1145 10.1007/s00520-012-1425-8 22402749

[B9] Brack E Bodmer N Simon A Leibundgut K Kühne T Niggli FK Ammann RA First-day step-down to oral outpatient treatment versus continued standard treatment in children with cancer and low-risk fever in neutropenia. A randomized controlled trial within the multicenter SPOG 2003 FN study Pediatr Blood Cancer 2003 2012 59 423 430 10.1002/pbc.24076 22271702

[B10] Vidal L Ben Dor I Paul M Eliakim-Raz N Pokroy E Soares-Weiser K Leibovici L Oral versus intravenous antibiotic treatment for febrile neutropenia in cancer patients Cochrane Database Syst Rev 2013 10 CD003992 10.1002/14651858.CD003992.pub3 PMC6457615 24105485

[B11] Higgins JPT Green S Cochrane Handbook for Systematic Reviews of Interventions 2008 Oxford: The Cochrane Collaboration

[B12] Centre for Reviews and Dissemination Systematic Reviews: CRD’s Guidance for Undertaking Reviews in Health Care 2009 York: CRD, University of York

[B13] NICE CG151 Neutropenic Sepsis: Evidence Review 2013 London: NICE

[B14] CG151 neutropenic sepsis: full guideline http://www.nice.org.uk/guidance/CG151

[B15] Feld R Paesmans M Freifeld AG Klastersky J Pizzo PA Rolston KVI Rubenstein E Talcott JA Walsh TJ Methodology for clinical trials involving patients with cancer who have febrile neutropenia: updated guidelines of the Immunocompromised Host Society/Multinational Association for Supportive Care in Cancer, with emphasis on outpatient studies Clin Infect Dis 2002 35 1463 1468 10.1086/344650 12471564

[B16] Haeusler GM Phillips RS Lehrnbecher T Sung L Ammann RA The reporting of outcomes in studies of fever and neutropenia in children with cancer: time for consensus Pediatr Blood Cancer 2013 60 1563 1564 10.1002/pbc.24662 23813563

[B17] National Institute for Health and Clinical Excellence Appendix J: Methodology Checklist: Prognostic Studies. From the Guidelines Manual 2009 London: National Institute for Health and Clinical Excellence

[B18] Phillips RS Lehrnbecher T Alexander S Sung L Updated systematic review and meta-analysis of the performance of risk prediction rules in children and young people with febrile neutropenia PLoS One 2012 7 e38300 10.1371/journal.pone.0038300 22693615 PMC3365042

[B19] Song F Parekh S Hooper L Loke Y Ryder J Dissemination and publication of research findings: an updated review of related biases Health Technol Assess 2010 14 234 10.3310/hta14080 20181324

[B20] Moher D Liberati A Tetzlaff J Altman DG PRISMA Group Preferred reporting items for systematic reviews and meta-analyses: the PRISMA statement PLoS Med 2009 6 e1000097 10.1371/journal.pmed.1000097 19621072 PMC2707599

