# Supplementary material for: Protocol for a systematic review of reductions in therapy for children with low-risk febrile neutropenia
Source: Syst Rev. 2014 Oct 21;3:119. doi: 10.1186/2046-4053-3-119 (PMC4234526; doi:10.1186/2046-4053-3-119)
Supplement: Additional file 1 — Appendix 1: Search strategy. Appendix 2: Email to elicit unpublished studies. Appendix 3: Study eligibility decision form. Appendix 4: Data extraction tool. Appendix 5: Assessment of risk of bias. Appendix 6: Anticipated statistical analyses. [file 2046-4053-3-119-S1.docx]

**Appendix 1 – Search Strategy**

Ovid MEDLINE(R) In-Process & Other Non-Indexed Citations and Ovid MEDLINE(R)

1. Agranulocytosis/ or neutropenia/ or leukopenia/
2. exp Fever/ or exp "Fever of Unknown Origin"/ or exp Body Temperature/
3. 1 and 2
4. (febrile adj5 (neutropen* or granulocytop* or agranulocyto* or leukocytop??ni*)).ti,ab.
5. ((fever or temperature or temp) adj5 (neutropen* or granulocytop* or agranulocyto* or leukocytop??ni*)).ti,ab.
6. 3 or 4 or 5
7. (antibiotic* or antimicrob* or anti-biotic* or anti-microb*).tw.
8. exp anti-bacterial agents/
9. exp beta-lactamases/ or exp beta-lactams/
10. exp penicillins/ or penicillin*.tw.
11. tazobactam*.tw.
12. ureidopenicillin*.tw.
13. exp ticarcillin/ or ticarcillin*.tw.
14. exp piperacillin/ or piperacillin*.tw.
15. exp quinolones/ or quinolone*.tw.
16. exp ciprofloxacin/ or ciprofloxacin*.tw.
17. exp ceftazidime/ or ceftazidime*.tw.
18. meropenem*.tw.
19. exp imipenem/ or imipenem*.tw.
20. exp aztreonam/ or astreonam*.tw.
21. exp aminoglycosides/
22. aminoglycoside*.tw.
23. exp amikacin/ or amikacin*.tw.
24. exp gentamicins/ or gentam?cin*.tw.
25. exp tobramycin/ or tobram?cin*.tw.
26. exp kanamycin/ or kanam?cin*.tw.
27. exp netilmicin/ or netilm?cin*.tw.
28. (beta-lactam* or beta?lactam*).tw.
29. or/7-28
30. exp "length of stay"/ or exp patient admission/ or exp patient discharge/ or exp patient readmission/ or exp inpatients/ or exp outpatients/
31. (discharge* or (length* adj2 stay*) or (duration* adj2 stay*) or admission* or readmission* or inpatient* or outpatient*).tw.
32. 30 or 31
33. 29 or 32
34. adolescent/ or exp child/ or exp infant/ or exp young adult/
35. (newborn* or new-born* or baby* or babies or neonat* or infan* or kid or kids or toddler* or adoles* or teen* or boy* or girl* or junevil* or youth* or puber* or prepuber* or pubescen*or prepubescen* or pediatric* or paediatric* or young person* or young people or young adult* or child* or schoolchild* or schoolage* or school* or preschool*).tw.
36. 34 or 35
37. 6 and 33 and 36

**Appendix 2 - Email to elicit unpublished studies**

Dear ­­______,

We are performing a systematic review into the care of children with low risk febrile neutropenia. In particular, we aim to investigate the safety and adequacy of oral antibiotics and/or early discharge compared with standard treatment. We are contacting you, as an expert within this field, to enquire if you know about any ongoing or unpublished work within this area. In particular, we are looking for studies that meet the following criteria:

Patients: Children or young adults (aged less than 18years) who attend paediatric services with fever and neutropenia and are assessed to be at low risk of medical complications.

Intervention (any of):

- Location of treatment: Inpatient, outpatient (less than 8 hours in hospital)
- Route of antibiotic administration: oral, IV

Study design: Controlled trials (randomised or quasi-randomised), or prospective single arm studies

Should you know of any appropriate studies, we would greatly appreciate if you could share this information. If you have any questions about whether a study might be eligible for inclusion within this systematic review, please do not hesitate to contact us.

Many thanks for your time and consideration,

Yours sincerely,

**Appendix 3 - Study Eligibility decision form**

1. Does the study include ≥ 80% children or young adults (aged less than 18years) who attend paediatric services with fever and neutropenia and are assessed to be at low risk of medical complications?

Yes Unclear No

2. Is the risk prediction rule clearly defined?

Yes Unclear No

3. Does the study assess any or the interventions/comparators defined in the protocol?

Yes Unclear No

4. Does the study assess any of the outcomes defined in the protocol?

Yes Unclear No

5. Is the study a controlled trial (randomised or quasi randomised) or prospective single arm study?

Yes Unclear No

6. Does the study enrol patients ≤24 hours after initial empiric treatment?

Yes Unclear No

Final decision:

Include Unclear (need more information – define what) No

**Appendix 4 - Data extraction tool**

**General information**

Person performing data extraction

Date of data extraction

Study number

Author

Title

Citation

Country of origin

Language

Source of funding

**Study information**

Aims/objectives of study

Study design

Inclusion criteria

Exclusion criteria

Definition of fever used

Definition of neutropenia used

Risk stratification tool used

Definition of low-risk within that tool

Timing of risk stratification

Details of randomisation/selection of cohorts

**Participants**

Number of participants

Age

Gender

Ethnicity

Socio-economic status (if given)

Disease

Are recruitment/refusal numbers given? If so, please record.

**Intervention and setting**

Description of intervention(s) (e.g. How long inpatient, if outpatient how often reviewed and where, antibiotics used, route, time of change)

**Outcome data/results**

Definition of treatment failure used

Outcomes measured

For each intervention(s) (as appropriate)

- Number of participants enrolled
- Number of participants included in analysis
- Number of withdrawals/exclusions/lost to follow-up
- Number of events for each of the primary and secondary outcomes in the study (provide details of each outcome given by the study)
- Number of events for each of the primary and secondary outcomes in this review (may be composite of outcomes provided by the study)

Details of patients who declined to consent (if given, number, distribution, reasons for declining given)

**Other information/Comments**

**Appendix 5 – Assessment of risk of bias**

**Randomised controlled trials** [11]

| **Domain** | **Support for judgement** | **Review authors’ judgement** |
| --- | --- | --- |
| *Selection bias.* |  |  |
| Random sequence generation. | Describe the method used to generate the allocation sequence in sufficient detail to allow an assessment of whether it should produce comparable groups. | Selection bias (biased allocation to interventions) due to inadequate generation of a randomised sequence. |
| Allocation concealment. | Describe the method used to conceal the allocation sequence in sufficient detail to determine whether intervention allocations could have been foreseen in advance of, or during, enrolment. | Selection bias (biased allocation to interventions) due to inadequate concealment of allocations prior to assignment. |
| *Performance bias.* |  |  |
| Blinding of participants and personnel *Assessments should be made for each main outcome (or class of outcomes).* | Describe all measures used, if any, to blind study participants and personnel from knowledge of which intervention a participant received. Provide any information relating to whether the intended blinding was effective. | Performance bias due to knowledge of the allocated interventions by participants and personnel during the study. |
| *Detection bias.* |  |  |
| Blinding of outcome assessment *Assessments should be made for each main outcome (or class of outcomes)*. | Describe all measures used, if any, to blind outcome assessors from knowledge of which intervention a participant received. Provide any information relating to whether the intended blinding was effective. | Detection bias due to knowledge of the allocated interventions by outcome assessors. |
| *Attrition bias.* |  |  |
| Incomplete outcome data *Assessments should be made for each main outcome (or class of outcomes).* | Describe the completeness of outcome data for each main outcome, including attrition and exclusions from the analysis. State whether attrition and exclusions were reported, the numbers in each intervention group (compared with total randomized participants), reasons for attrition/exclusions where reported, and any re-inclusions in analyses performed by the review authors. | Attrition bias due to amount, nature or handling of incomplete outcome data. |
| *Reporting bias.* |  |  |
| Selective reporting. | State how the possibility of selective outcome reporting was examined by the review authors, and what was found. | Reporting bias due to selective outcome reporting. |
| *Other bias.* |  |  |
| Other sources of bias. | State any important concerns about bias not addressed in the other domains in the tool.  If particular questions/entries were pre-specified in the review’s protocol, responses should be provided for each question/entry. | Bias due to problems not covered elsewhere in the table. |

**Single arm studies** [17]

1. The study sample represents the population of interest with regard to key characteristics, sufficient to limit potential bias to the results

Yes No Unclear

1. Loss to follow-up is unrelated to key characteristics (that is, the study data adequately represent the sample), sufficient to limit potential bias

Yes No Unclear

1. The prognostic factor of interest is adequately measured in study participants, sufficient to limit potential bias

Yes No Unclear

1. The outcome of interest is adequately measured in study participants, sufficient to limit bias

Yes No Unclear

1. Important potential confounders are appropriately accounted for, limiting potential bias with respect to the prognostic factor of interest

Yes No Unclear

1. The statistical analysis is appropriate for the design of the study, limiting potential for the presentation of invalid results

Yes No Unclear

**Appendix 6 – Planned statistical analyses**

**Main analyses**

**Comparative (RCTs and quasi-randomised studies)**

- Oral vs intravenous antibiotics

Treatment failure/ safety/ adequacy (odds ratios)

- Outpatient vs inpatient treatment

Treatment failure/ safety/ adequacy (odds ratios)

**Non-comparative (RCTs, quasi-randomised studies and prospective, single arm studies**

- Route of administration of antibiotics

Treatment failure, safety and adequacy (weighted mean)

- Location of care

Treatment failure, safety, adequacy and readmission rate (weighted mean)

- Declining to consent for outpatient treatment (either as randomisation or in a single arm study) (weighted mean)

**Subgroup analysis**

**Comparative**

- Location of treatment

Stratified by time of discharge (odds ratio)

- Location of treatment

Stratified by risk prediction tool used (odds ratio)

**Non-comparative**

- Outpatient care
  - Stratified by time of discharge (entirely outpatient, <24 hours, 24-48 hours, >48 hours) (weighted mean)
  - Stratified by risk prediction tool used (weighted mean)
  - Stratified by timing of risk stratification (weighted mean)

**Sensitivity analysis**

- Full reports only
- RCTs only
- Risk of selection bias and attrition bias
- Use of fixed effect meta-analysis
- Definition of fever and neutropenia used
- Definition of treatment failure used
- Antibiotics used
- Developing world compared with developing world
